# Knowledge and practices of general surgeons and residents regarding spilled gallstones lost during laparoscopic cholecystectomy: a cross sectional survey

**DOI:** 10.1186/1754-9493-7-27

**Published:** 2013-08-14

**Authors:** Muhammad Sohaib Khan, Muhammad Adil Khatri, Muhammad Shoaib Khan, Zakiuddin G Oonwala

**Affiliations:** 1 Civil Hospital, Baba-e-urdu Road, Karachi, Pakistan; 2 Hamdard University Hospital, M.A. Jinnah Road, Karachi, Pakistan

## Abstract

**Background:**

Gall bladder perforation, gallstone spillage and loss are commonly reported from Laparoscopic Cholecystectomy (LC). Though rare, lost gallstones can cause a variety of complications presenting variably from within 1 month to 20 years postoperatively. Our objective was to investigate knowledge and practices of surgeons and surgical residents regarding spilled gallstones lost during laparoscopic cholecystectomy.

**Methods:**

An observational, cross-sectional survey, using a questionnaire based on 13 self-answered close-ended questions, was conducted at 6 different post-graduate centers in Karachi, Pakistan.

**Results:**

Of the 82 participants, 23 (28%) were consultant surgeons while 59 (72%) were general surgery residents. 86% of participants were aware that stones lost during LC can cause complications. Out of the 18 reported complications presented, only 20% participants identified more than 8 complications for which they can consider lost gallstones causal. 28% of participants weren’t aware about the expected postoperative duration for presentation of complications. Only 15% of our participants expected complications beyond 5 years of the procedure. 72% of participants will not convert to open cholecystectomy to retrieve lost gallstones. While 88% of participants agreed that lost gallstones should be documented in operative notes, only 70% reported that it’s actually done in practice. 55% of participants agreed to have possibility of lost gallstones as part of the informed consent but in practice it’s included according to only 31% of participants. 68% of participants believe that patients should be informed if gallstones are lost but in actual practice only 41% participants inform patients when gallstones are lost during procedure.

**Conclusions:**

We conclude that there is a dearth of awareness regarding diversity of complications from lost gallstones and about their variable postoperative duration of presentation. The practices involving lost gallstones management, documentation and patient information were found to vary widely. Proper awareness is imperative as it may compel surgeons to undertake all possible measures to retrieve spilled gallstones and progress towards better and standardized practices in managing lost gallstones.

## Introduction

Laparoscopic cholecystectomy (LC) is a commonly performed procedure in general surgical units all over the world. The inherent advantages of the procedure that include low postoperative morbidity with a significant economic impact were recognized after few years of its introduction. In 1992, the consensus statement from the National Institute of Health (NIH) Conference accepted LC as the treatment of choice for patients with symptomatic gallstones [1].

Like any other procedure, LC has known complications of which iatrogenic perforation and gallstone spillage are more commonly reported. The incidence of gall bladder perforation during LC has been reported to be up to 36% [2] with reported spillage of gallstones as high as 16% [3]. Though rare, the gallstones spilled during LC if left unretrieved can result in serious complications [4]. A review of the Medline database for reports on lost gallstones during LC found septic, fistulous and even intestinal complications with variable presentations in a wide variety of locations [5]. These complications have been reported to present within a month to up to 20 years after the procedure [3,6].

There is a lack of consensus on the management of spilled gallstones lost during LC. For some surgeons the rarity of complications dismisses the prospects of converting the LC to an open procedure for retrieving the spilled stones, while for others the seriousness of complications demands that every effort be made to retrieve all gallstones spilled during the procedure. Mullerat [7] reported that only a fifth of surgeons include this phenomenon in the informed consent and only half the surgeons inform the patients in case the stones are lost during LC. They observed that informing the patients may aid in the diagnosis of the complications if in case they occur, but can also result in unnecessary anxiety and repeated examinations for rare complications.

It is clear that awareness of the possible complications of this condition amongst the surgical team is necessary for making an informed decision about its timely management. Our objective was to investigate the knowledge and practices of surgeons and surgical residents regarding spilled gallstones lost during LC in post graduate training centers of Karachi, Pakistan.

### Methodology

This was an observational, cross-sectional survey with participants belonging to 6 different post-graduate centers in Karachi, Pakistan. The centers included Dow University of Health Sciences, Hamdard University Hospital, the Aga Khan University Hospital, Abbasi Shaheed Hospital, Liaquat National Postgraduate Medical Center and the Indus Hospital.

The participants included practicing surgeons and surgeons at different levels of training and a convenient sample was obtained.

### The questionnaire

The survey consisted of 13 self-answered close-ended questions. The questionnaire covered 5 aspects of spilled gallstones lost during laparoscopic cholecystectomy including: 1) knowledge of occurrences, 2) knowledge of complications, 3) management, 4) documentation and 5) patient information. The questionnaire was divided into 2 parts to assess attitudes and practices of the participants. The questionnaire was validated and standardized by conducting a pilot study on 10 participants.

## Results

Of the 82 participants, 23 (28%) were consultant surgeons while 59 (72%) were residents of general surgery at different post-graduate training levels. Overall, 47 (57%) participants were from public-sector hospitals and 35 (43%) from private-sector teaching hospitals.

### Knowledge

**Figure 1:**
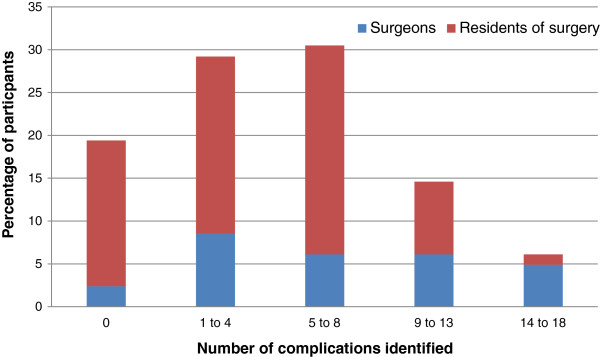
**Presented complications from lost gallstones as in Table **1 **identified as causal.**

**Figure 2:**
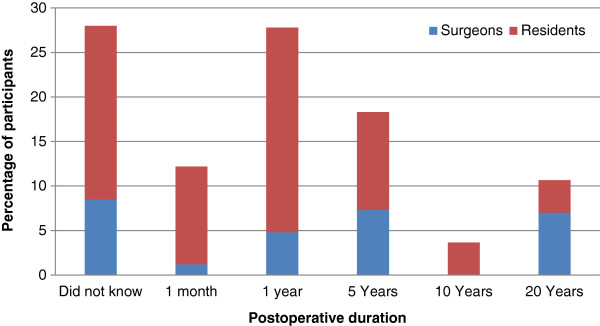
Expected postoperative duration for presentation of complications.

Majority (86.5%) of the participants had experience with lost gallstones during LC. Responding to the question of prevalence of lost gallstones in LC, 60% participants held it was less than 5%. In response to the question regarding vulnerability of technique, 43% considered both single and multiple port to be equally vulnerable while 24% considered single port to be more vulnerable. Up to 83% of respondents were aware of the possibility of complications from lost gallstones as opposed to 10% of our participants who believed that lost stones cannot cause complications. Out of the 18 reported complications presented (Table 1), participants were to identify complications that they can consider causal from lost gallstones (Figure 1). 20% of the participants identified more than 8 complications, 60% of participants identified less than 9 complications while 20% of participants did not identify any complication. When asked about the duration post LC at which complications can present (Figure 2), 28% of participants were not aware about the expected duration. 30% of participants expect complications for a year after the procedure while 17% expect complications for 5 years. Only 15% expect complication beyond five years of the procedure.

**Table 1:** List of reported complications presented to survey participants with references

Sinus formation [8]	Bladder obstruction [9]
Dyspareunia [10]	Empyema [11]
Colocutaneous fistula [12]	Subhepatic abscess [13]
Cholelithoptysis [14]	Liver abscess [15]
Pelvic abscess [16]	Hematuria [17]
Septicimia [18]	Intra-abdominal abscess [19]
Retroperitoneal abscess [20]	Intestinal Obstruction [21]
Granuloma formation [22]	Loin abscess [23]
Incarcerated hernia [24]	Port site infection [25]

### Attitude and practice

In case spilled stones are lost, 72% of survey participants will not convert LC to open cholecystectomy, though 18% of the participants will convert to open in case gallstones are lost while 7% of participants did not know. Documentation of lost gallstones in operative notes should be done according to 88% of participants but only 70% of participants said that it is actually documented while according to 9% of participants, documentation of lost gallstones is done ‘sometimes’. According to 16% of participants lost gallstones are never documented.

**Figure 3:**
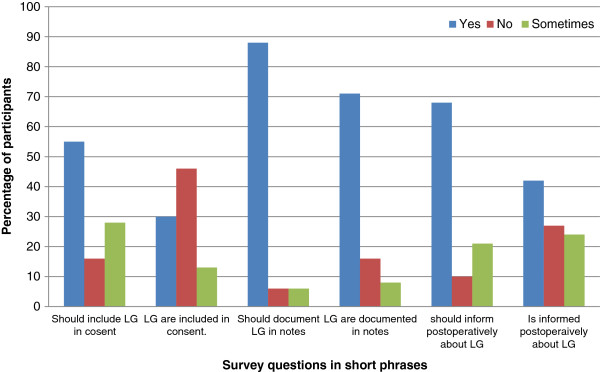
Patient information and documentation of lost gallstones (LG).

Regarding aspects of patient counseling, 55% participants responded that the patient should be informed prior to surgery as part of the informed consent regarding possibility of lost gallstones. However, 47% participants reported that patients are never informed prior to surgery in actual practice, while 31% believed that patients are informed. Postoperatively, 68% participants believe that patients should be informed if gallstones are lost. When asked about current practice, 41% participants informed patients of lost gallstones postoperatively, while 27% participants don’t inform patients. According to 24% of participants, patients are informed ‘sometimes’ (Figure 3).

## Discussion

Laparoscopic Cholecystectomy (LC), since its recognition as the treatment of choice for symptomatic cholelithiasis, has become one of the most commonly performed general surgical procedures. The laparoscopic technique, due to lower incidence of postoperative morbidity and early recovery, has become the gold standard for performing cholecystectomies [1]. Like all other surgical interventions, LC has its known complications, of which bile duct injury is the most serious and dreaded complication. Fortunately the rate of this serious complication has been reported to be as low as 0.2% [26]. Gall bladder perforation and gall stone spillage are the other more common complications of LC. The incidence of gall bladder perforation during LC has been reported to be up to 36% [2] with reported spillage of gallstones as high as 16% [3]. Spilled gallstones are also lost during LC being reported to have occurred in 1.1% cases by Diez [27]. In our survey majority (86.5%) of the participants agreed that gallstones are lost during LC. 60% of participants believed that stones are lost in up to 5% of LCs while according to 23% gallstones are lost in more than 5% of cases which is much greater than the percentage reported in the literature.

Spilled gallstones that are lost during LC rarely cause complications yet up to 83% of respondents of our survey were aware of the possibility of complications from lost gallstones as opposed to 10% who believed that lost stones cannot cause complications. A wide variety of complications presenting over a variable period post LC, have been reported in the literature. Abscesses are most frequently reported. These include intra-abdominal abscess [19], retroperitoneal abscess [20], pelvic abscess [16], loin abscess [23], subhepatic abscess [13] and liver abscess [15]. The diverse locations of these abscesses attest to the migratory nature of lost gallstones. This may also be the reason for many other varied complications that have been reported including colocutaneous fistula [12], intestinal obstruction [21], bladder obstructions [9], empyema [11], cholelithoptysis [20], septicemia [18], incarcerated hernia [24] and dyspareunia [10]. Out of the 18 reported complications from lost gallstones that were presented to the participants of our survey (Table 1), only 20% of the participants identified more than 8 complications for which they can consider lost gallstones causal. This shows that even though most of the participants of our survey aware about the possibility of complications from lost gallstones, a vast majority isn’t aware about the variable nature of these complications.

Complications from lost gallstones present at variable durations postoperatively ranging from with a month to as much as 20 years post operatively [3,6]. In our survey, 28% of participants were not aware about the expected duration elaborating the dearth of awareness regarding this aspect. The expectations of only 15% of our participants were in accordance with the reported variability of postoperative duration of presentation as they expected complications even beyond 5 years of the procedure.

The rarity of complications from lost gallstones has raised the question that should patients be informed prior to surgery about the possibility of such an event. In a survey conducted by Mullerat [7], 80% of surgeons don’t have possibility of lost gallstones as part of informed consent. In our survey 55% participants agreed that the patient should be informed prior to surgery regarding possibility of lost gallstones as part of the informed consent. However, 47% participants reported that patients are never informed prior to surgery in actual practice. In Pakistani medical practice, this split opinion also conforms to the already reported uncertainty of our medical practitioners regarding the amount information deemed appropriate for the patient with illiteracy of the patient being the major influence [28]. Prior to elective surgeries, patients in Pakistan have been reported to have very high levels of anxiety with fear of complications being the major reason behind it [29]. Proper counseling with clear understanding of incidences of various complications by the surgical team is essential as it will be instrumental in enhancing the patients’ confidence ameliorating their anxiety.

Moreover, conversion to open cholecystectomy for retrieval is not indicated as the benefits of the laparoscopic technique outweigh the threat posed by lost gallstones as they are very rare being reported in 0.08% to 0.58% cases of lost gallstones [3,30]. Yet the serious nature of these complications demands that every effort be made to retrieve spilled gallstones. 72% of our survey participants will not convert LC to open cholecystectomy. This indicates the unclear state of 28% our participants regarding the intra operative management of lost gallstones.

In the event that lost gallstones are lost, informing the patient presents as a predicament to the surgeon as on one hand it is necessary so that it may facilitate diagnosis of any future complications but such information may result in unnecessary anxiety to the patient. Postoperatively, 68% participants of our survey believe that patients should be informed if gallstones are lost. When asked about current practice, 41% participants informed patients of a lost gallstone postoperatively, while 27% respondents never informed patients. According to 24% of participants, patients are informed ‘sometimes’. This split opinion regarding informing patients about lost gallstones has also been reported by Mullerat [7] with only 50% of surgeons actually informing patients in their survey.

In all instances, lost gallstones should be documented in operative notes as it may not only facilitate diagnosis of the resultant complications but will also allow an objective assessment of the incidence of lost gallstones. In our survey, documentation of lost gallstones in operative notes should be done according to 88% of participants but only 70% of participants report that it is done in actual practice while according to 9% of participants, documentation of lost gallstones is done ‘sometimes’. According to 16% of participants lost gallstones are never documented. Wauben [31] has identified the inadequacy of operative notes of laparoscopic cholecystectomy being representative of the procedure. Our survey too has established that even lost gallstones are often not documented in operative notes elaborating their inadequacy further. From this revelation we can also anticipate that estimating the frequency of lost gallstones from operative notes alone will be underreporting the actual number of cases leading to misinterpretation of the practices at large.

## Conclusion

We conclude that there is a dearth of awareness regarding the diversity of complications from lost gallstones as well as about their variable postoperative duration of presentation. The practices involving lost gallstones management, documentation and patient information were found to vary widely. With the revelation from our survey that lost gallstones are often not documented in the operative notes, we can anticipate that any estimation of the frequency of lost gallstones from operative notes alone will be under reporting the actual cases potentially leading to misinterpretation of the practices at large.

Opinions and practices of our survey participants were found to be specially divided in aspects of pre-operative consent and postoperative information for the patient. We recommend further investigation to ascertain the reasons behind this split opinion as then steps may be taken to promote more standardized evidence based practices allowing the patient to be fully informed about all possible predicaments that may ever arise without provoking any unnecessary anxiety.

Proper awareness of the surgical team regarding lost gallstones is imperative as it may then compel surgeons to undertake all possible measures to retrieve spilled gallstones and progress towards better and standardized practices involving lost gallstones ensuring safer surgeries and allowing prompt recognition of complications if ever they arise.

## Competing interests

The authors declare that they have no competing interests.

## Authors’ contributions

MS (corresponding author) conceived and designed the project, identified its significance through literature search, designed the questionnaire, conducted the survey, analyzed and interpreted the data and wrote the manuscript. MA contributed to the literature search, designing of questionnaire, conducted the survey and was involved in analysis and interpretation of the data. MSK assisted the designing of questionnaire, provided statistical expertise in analysis and interpretation of data and assisted in writing of manuscript. ZGO supervised the whole project providing expert opinion and guidance on designing the questionnaire, facilitated the survey, provided critical insight on interpretation of data and also supervised the writing of manuscript. All authors read and approved the final manuscript.
